# Engineering noise control for mines: Lessons from the world

**DOI:** 10.4102/sajcd.v67i2.684

**Published:** 2020-03-31

**Authors:** Milka C. Madahana, Otis T. Nyandoro, Nomfundo F. Moroe

**Affiliations:** 1 School of Electrical and Information Engineering, Faculty of Engineering, University of the Witwatersrand, Johannesburg, South Africa; 2 Department of Speech Pathology and Audiology, Faculty of Humanities, University of the Witwatersrand, Johannesburg, South Africa

**Keywords:** Noise, Engineering, Barriers, Isolators, Controls

## Abstract

**Objective:**

The main objective of this article was to present some of the engineering noise control methods that are currently being used across the world in the mining industry, while at the same time interrogating noise control measures that could be applied to effectively reduce noise emissions from the equipment utilised within South African mines.

**Background:**

A brief summary of the hearing conservation programmes used in South Africa is presented. Summarised research on the use of engineering noise control in South Africa is also presented, with an overview of the various engineering noise control methods applied across the world in dealing with occupational noise.

**Method:**

For illustrative purposes, case studies were used to show how engineering noise controls could be used to reduce the noise levels and risks within this context.

**Results:**

Some of the case studies used have cited a reduction in the noise intensity emitted by machinery from a range of 93 dBA – 104 dBA to a range of 90 dBA – 94 dBA, demonstrating quite a significant reduction in the noise emission of the equipment. This article further provides recommendations on how South African mines could contextualise these methods.

**Conclusion:**

One of the key recommendations is encouraging the South African mining industry towards the documenting and publishing of those engineering noise control methodologies that have proven to be effective for shared best practice. A need was identified for extensive research to be conducted and documented evidence to be made available to assist the South African mining industry with locating and assessing current engineering controls available in South Africa. Machines and processes that require noise control should be identified and, lastly, the current barriers to the use of engineering noise control methodologies should be identified, with the main goals of finding ways to overcome the noise challenges in the mines.

## Introduction

Noise may be defined as an undesirable sound lacking musical quality (Concha-Barrientos, Campbell-Lendrum, & Steenland, 2004). Depending on the sources from which it is generated, noise can be classified as intermittent, impact or impulsive noise, repetitive impact noise, continuous narrowband noise and continuous wide band noise (Concha-Barrientos et al., 2004). These classifications of noise are emitted in the mines at varying frequencies and intensities (Basner et al., 2014). Excessive occupational noise levels and exposure to such noise have been documented to cause occupational noise-induced hearing loss (ONIHL), tinnitus, disturbance in speech communication and increased risks of accidents (Lie et al., 2016; Lopes, Otowiz, Lopes, Lauris, & Santos, 2012).

Occupational noise-induced hearing loss is one of the most prevalent occupational diseases caused by extended exposure to loud noise (Nanda, 2012). Hearing conservation programmes (HCPs) were introduced to the South African mines by the Leon Commission of Enquiry in 1994 (Stanton, 2003). This was one of the measures recommended to deal with the noise dilemma in the South African mining industry.

## A brief summary of hearing conservation programme pillars

**FIGURE 1:**
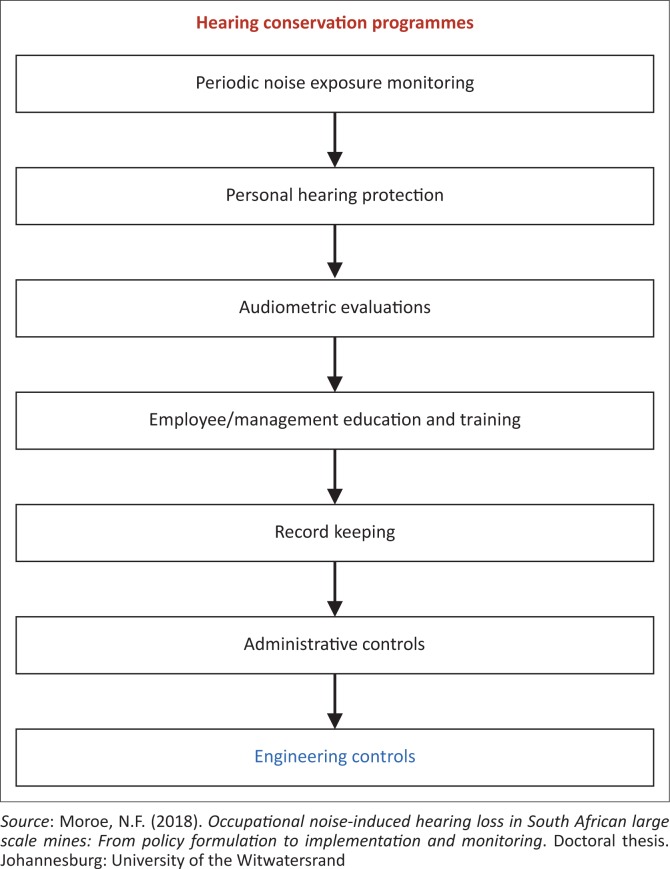
Seven pillars of an effective hearing conservation programme.

The seven pillars of HCPs as documented by Moroe, Khoza-Shangase, Madahana and Nyandoro (2019) are shown in Figure 1.

This article mainly focuses on the last pillar shown in Figure 1 (i.e. engineering controls), which entails the use of noise controls. The main objective of this article was to present some of the engineering noise control methods that are currently being used across the world in the mining industry and how these noise control measures can be applied effectively to reduce noise emissions from machines in the South African mines. This article is structured as follows: a brief introduction is given, followed by the subsections listed below that show how the main objective will be achieved:

1. A brief summary of hearing conservation programme (HCP) pillars
2. Overview of engineering noise control methods
3. Summarised research on the use of engineering noise control in South Africa
4. For illustrative purposes, Case studies of engineering noise control methods currently being used across the world are used to show how engineering noise controls can be used to reduce noise levels in this context.
5. Recommendations on how South African mines can contextualize the noise control methods used by the rest of the world.

## Overview of engineering noise control methods

**FIGURE 2:**
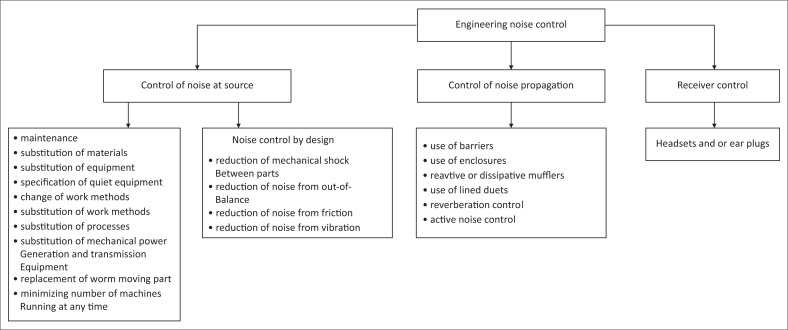
An overview of engineering noise controls.

The engineering noise control methods can be divided into: Control of noise at source, control of noise propagation and receiver control as shown in Figure 2.

## Summarized research on the use of engineering noise control in South Africa

Gumede, Blomerus, Coutts and De Beer (2014) conducted a study that proposed a strategy to buy quitter equipment, which is one of the engineering noise control methods. It is in the category of the control of noise at source as shown in Figure 2. The findings of the research indicated that the mining industry perceives the buying of quitter equipment as a proactive strategy that will aid in the reduction of ONIHL, thus improving the management of noise from source. Burger, Von Wielligh, De Wet, Otterman and Steyn (2004) developed a low noise blast-hole drilling system whose main goal was to reduce the risks associated with the exposure to excessive noise in the underground mining. The system was tested in an underground mining environment.

## Case studies of engineering noise control methods currently being used across the world

### Identification of machinery that emits the most noise in the mines

Sensogut (2007) documented the main sources of noise in the mine to be excavators, stage loaders, shearers, compressors, fans, continuous miners, pneumatic drilling machines, vibrating screens, rotating breakers and mills. Spencer (2009) also carried out an investigation on equipment operator’s noise exposure in western underground gold and silver mines. The research findings on some of the equipment that emit the loudest noise were consistent with Sensogut’s (2007) findings. Effective engineering noise controls entail understanding the production of quieter processes by understanding the noise producing mechanism. It is much easier when the implementation is done in the original design. Most engineers do not have access to the equipment and can only apply extra features to the machines once they have been purchased. These additional features can sometimes be cumbersome and perceived as an obstruction to progress at work.

### Noise mapping

Case studies show how engineering noise controls can be used to reduce noise levels in this context. Nikola, Aleksandar, Dinko, Vladimir and Uros (2018) proposed that in order to have a mine with successful noise management, there is a need for an assessment to be carried out in the initial stages of setting up the mine to establish in advance that the mining activities will have reasonable noise effects. It is further proposed in the paper that the following measures should be taken at the design phase of the mine: noise mapping of the mine should be carried using efficient and accurate computer modelling software (Nikola et al., 2018), baseline characterisation should also be performed to understand the characteristics of the area in which the mine is located. Noise emission estimation for each machinery should be done using available international standards to estimate and quantify the anticipated noise that will be coming from each unit and the entire mine. The noise emission estimation should be done in conjunction with the manufacturer’s data sheet and the actual measurements (Nikola et al., 2018). The research further proposes that noise assessment and control should always be one step ahead of the potential problem.

### Control of noise at source and noise propagation

The research by Sensogut (2007), using a case example of mines in Turkey, indicates that noise controls and administrative actions should always be ranked as the first line of defence when it comes to combating noise dilemma in the mines. The research further suggests that engineering noise control measures to be implemented would include the following: selecting of processes with lower sound pressure level, locating of a mine in noiseless places, enclosing the noise sources and prevention of noise from being transmitted and moving of noisy machines to sections of the mine that are used less (Sensogut, 2007).

The Peterson (2018) proposed that research (Peterson, 2018) proposed that engineering controls require physical changes to the workplace, such as redesigning of equipment to eliminate noise sources and constructing barriers that prevent noise from reaching a worker. Manwar, Mandal and Pal (2016) carried out research which was based on using noise mapping as a measure obtained from the European mines to combat the noise dilemma. The findings of the research indicated that prediction of total amount of noise likely to be emitted by a mine can be used as a tool for understanding the type of noise measures to be taken to minimise the effects of noise on the miners and surrounding communities. The research further proposed the use of engineering control methods, for example, using acoustic barriers near machines that emit excessive noise or crushers to attenuate noise propagation. Reeves, Randolph, Yantek and Peterson (2009) carried out a research in noise control in underground metal mining. In this work, it is proposed that some of the lessons learnt that could aid in the reduction of noise in underground mines were correct use of sound barriers; for high frequencies, it is recommended that environmental cabs and windshield should be used. Plugging gaps in machine panels and wind shields combined with a material that can create an airtight seal that can also be used to create barriers. Checking that there are no gaps in barriers because they compromise the effectiveness of noise control and in a situation where enclosures are necessary, a partial enclosure can be provided and can be used. It is further recommended that enclosures should be lined with absorptive materials that are thick enough to absorb the dominant sound frequencies. Spencer and Reeves (2010) conducted a research on the assessment of engineering noise controls at a talc-processing plant and found a reduction in sound level from a range of 93 dBA – 104 dBA to a range of 90 dBA – 94 dBA. The engineering control measures that were used to achieve this reduction were placing of curtains around fans and the crushers. The mill was treated with a ductwork with a sound barrier material.

### Control of noise at source by design

Peterson, Miller and Yantek (2014), working with the National Institute for Occupational Safety and Health (NIOSH), visited a collaborating mine and investigated a haul truck underground in a shop area using Source Path Contribution (SPC) techniques for Noise Source Identification (NSID) (Peterson et al., 2014). Further studies were conducted in 2018, using dosimetry and time motion to determine when an operator accumulates the most noise dose (Peterson, 2018). The investigations carried out by Peterson involved retrofitted noise control package for load haul dumps (LHDs) used in underground metal and non-metal mines. The results showed that the reduction in LHD operator exposure was less than that of the haul truck operator. A reduction of 2 dB – 9 dB was achieved. Saleh, Woskie and Bello (2016) conducted a research on installation of acoustic curtains on fans and crushers for noise reduction and found that there was a noise reduction from a range of 93 dB – 104 dB to a range of 88 dB – 94 dB. Camargo, Azman and Alcorn (2016) carried out a research that was targeting the reduction of noise emitted by the long wall shearer cutting drums. Several models were built and tested before choosing structural modification to increase the stiffness of the outer vane plates as a suitable method. This method was implemented and a reduction of 3 dB was achieved.

## Recommendations on how South African mines can contextualise the noise control methods used by the rest of the world

Engineering noise controls that have been discussed may be implemented in the South African mines, for example, by enclosing noisy places, using correct sound barriers and using curtains and retrofitted noise control packages. There is limited published evidence from the South African mines that document how engineering noise controls have contributed towards a reduction in noise emission from equipment. Mining companies and research centres in South Africa should consider partnering with NIOSH because of their extensive documented research work on ONIHL in the mines, to extend and contextualise their research to South African mines. The South African mines should thereafter consider conducting research and the documented evidence should be made available to assist with locating and assessing current engineering controls in South African mines. This research work should also include identification of machines and processes that require noise control. Current barriers to the use of noise control should be identified with the main goals of finding ways to mitigate them.

## Limitations of the work and conclusion

This article is not a systematic review paper; hence, not all engineering noise control methods currently have been applied. The engineering noise control method as a technique to mitigating noise in the mines has been presented. Case studies from around the world and how they can be contextualised to the South African mining industry have also been presented. A follow-up to this work would include the documentation of a strategic plan that would encourage the mining industry to publish the noise control interventions that have currently worked for them.
